# Crystal structure and Hirshfeld surface analysis of 4-(2-chloro­eth­yl)-5-methyl-1,2-di­hydro­pyrazol-3-one

**DOI:** 10.1107/S2056989024000835

**Published:** 2024-01-31

**Authors:** Farid N. Naghiyev, Victor N. Khrustalev, Mehmet Akkurt, Evgeny A. Dukhnovsky, Ajaya Bhattarai, Ali N. Khalilov, İbrahim G. Mamedov

**Affiliations:** a Department of Chemistry, Baku State University, Z. Khalilov str. 23, Az, 1148, Baku, Azerbaijan; b Peoples’ Friendship University of Russia (RUDN University), Miklukho-Maklay St. 6, Moscow 117198, Russian Federation; c N. D. Zelinsky Institute of Organic Chemistry RAS, Leninsky Prosp. 47, Moscow, 119991, Russian Federation; d Department of Physics, Faculty of Sciences, Erciyes University, 38039 Kayseri, Türkiye; e Department of Chemistry, M.M.A.M.C (Tribhuvan University) Biratnagar, Nepal; f "Composite Materials" Scientific Research Center, Azerbaijan State Economic University (UNEC), H. Aliyev str. 135, Az 1063, Baku, Azerbaijan; Institute of Chemistry, Chinese Academy of Sciences

**Keywords:** crystal structure, hydrogen bonds, dimers, pyrazole ring, Hirshfeld surface analysis

## Abstract

In the crystal of the title compound, C_6_H_9_ClN_2_O, mol­ecular pairs form dimers with an *R* ^2^_2_(8) motif through N—H⋯O hydrogen bonds. These dimers are connect into ribbons parallel to the (100) plane with *R* ^4^_4_(10) motifs by N—H⋯O hydrogen bonds along the *c-*axis direction. In addition, π–π [centroid-to-centroid distance = 3.4635 (9) Å] and C—Cl⋯π inter­actions between the ribbons form layers parallel to the (100) plane. The three-dimensional consolidation of the crystal structure is also ensured by Cl⋯H and Cl⋯Cl inter­actions between these layers. According to a Hirshfeld surface study, H⋯H (43.3%), Cl⋯H/H⋯Cl (22.1%) and O⋯H/H⋯O (18.7%) inter­actions are the most significant contributors to the crystal packing.

## Abstract 2

In the crystal, mol­ecular pairs form dimers through N—H⋯O hydrogen bonds. These dimers are linked into ribbons parallel to the (100) plane by further N—H⋯O hydrogen bonds. In addition, π–π and C—Cl⋯π inter­actions between the ribbons form layers parallel to the (100) plane.

## 1. Chemical context

**Figure 1:**
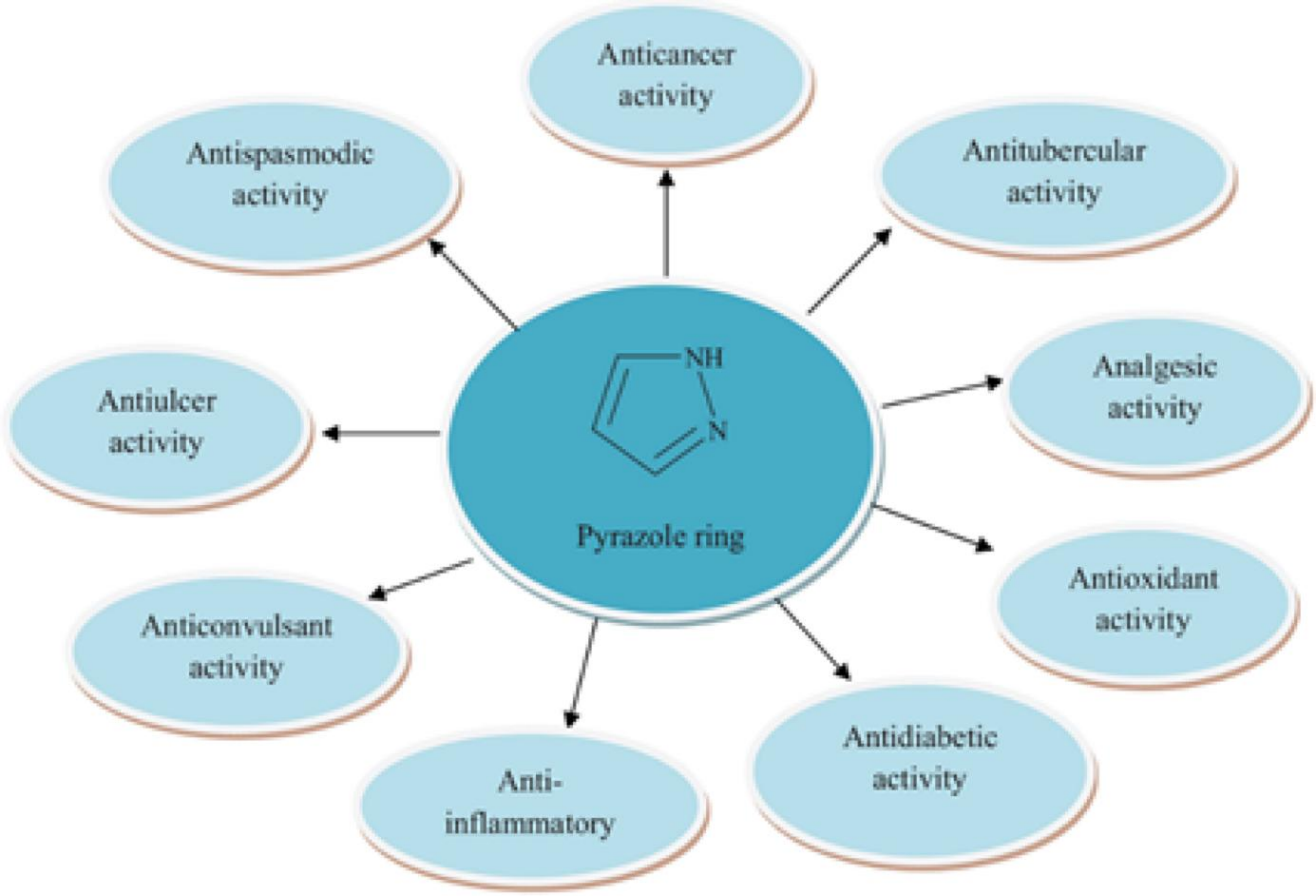
The biological activities of compounds incorporating the pyrazole motif.

Nitro­gen-based heterocyclic compounds are an important branch of organic chemistry. These systems have received increasing attention over the past two decades. Synthetic chemistry is growing extensively with recently developed heterocyclic systems for various research and commercial purposes (Maharramov *et al.*, 2021 ▸, 2022 ▸; Erenler *et al.*, 2022 ▸; Akkurt *et al.*, 2023 ▸). These systems have found wide applications in diverse branches of chemistry, including the chemistry of coordination compounds (Gurbanov *et al.*, 2021 ▸; Mahmoudi *et al.*, 2021 ▸), drug development (Donmez & Turkyılmaz, 2022 ▸; Askerova, 2022 ▸) and material science (Velásquez *et al.*, 2019 ▸; Afkhami *et al.*, 2019 ▸). The pyrazole motif is the most widespread five-membered heteroaromatic ring system in nitro­gen heterocycles. It is an essential structural motif present in many natural bioactive mol­ecules such as l-α-amino-β-(pyrazolyl-*N*)-propanoic acid, withasomnine, pyrazofurin, pyrazofurin B, formycin, formycin B, oxoformycin B, nostocine A, fluviols (A, B, C, D and E), pyrazole-3(5)-carb­oxy­lic acid, 4-*M*ethyl pyrazole-3(5)-carb­oxy­lic acid, 3-*n*-nonyl­pyrazole (Khalilov *et al.*, 2022 ▸; Kumar *et al.*, 2013 ▸; Sobhi & Faisal, 2023 ▸). The pyrazole ring incorporating derivatives with various biological activities (Singh *et al.*, 2023 ▸), such as anti­convulsant, anti­diabetic, anti-inflammatory, anti­oxidant, anti­cancer, anti­tubercular, anti­ulcer activities and other properties has been reviewed recently (Fig. 1 ▸).

**Figure 2:**
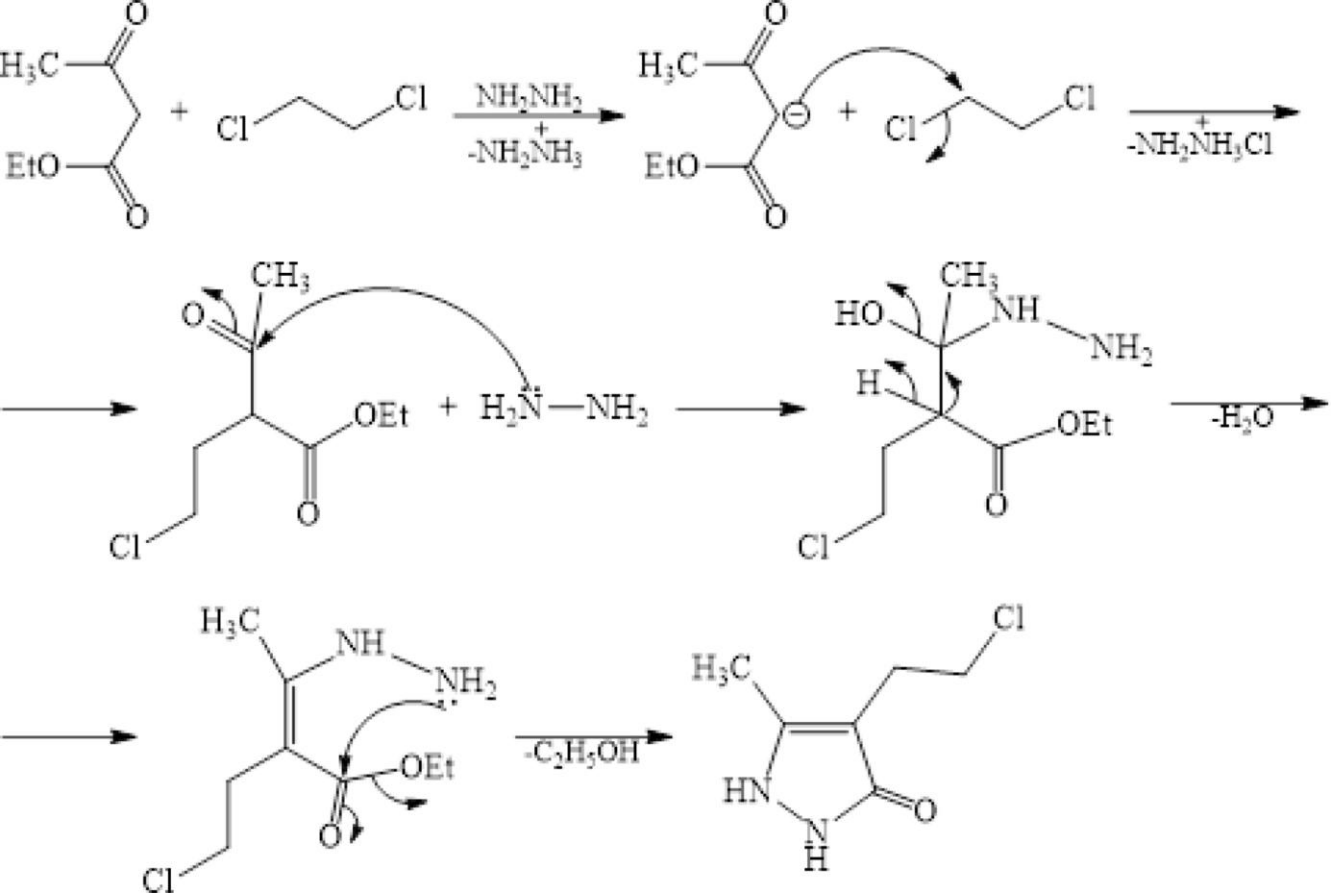
The proposed reaction mechanism for the formation of the title compound.

On the other hand, the incorporation of various pharmacophore groups in a pyrazole scaffold has led to the development of best-selling drugs such as ibrutinib, ruxolitinib, axitinib, niraparib and baricitinib (Atalay *et al.*, 2022 ▸; Alam, 2023 ▸). Thus, in the framework of our studies in heterocyclic chemistry (Naghiyev *et al.*, 2020 ▸, 2021 ▸, 2022 ▸), we herein report the crystal structure and Hirshfeld surface analysis of the title compound, 4-(2-chloro­eth­yl)-5-methyl-1,2-di­hydro­pyrazol-3-one, for which the proposed reaction mechanism is shown in Fig. 2 ▸. 
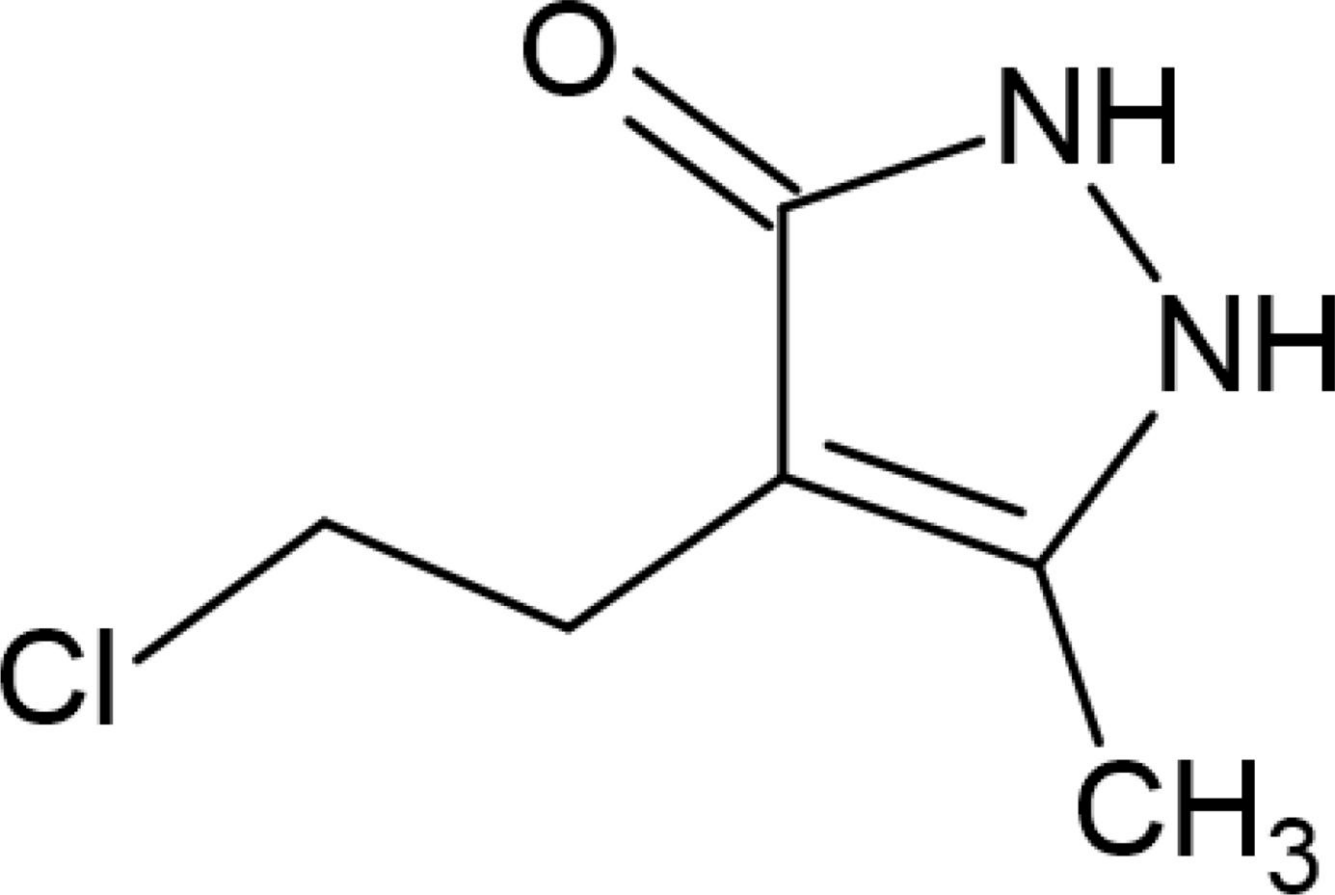


## 2. Structural commentary

**Figure 3:**
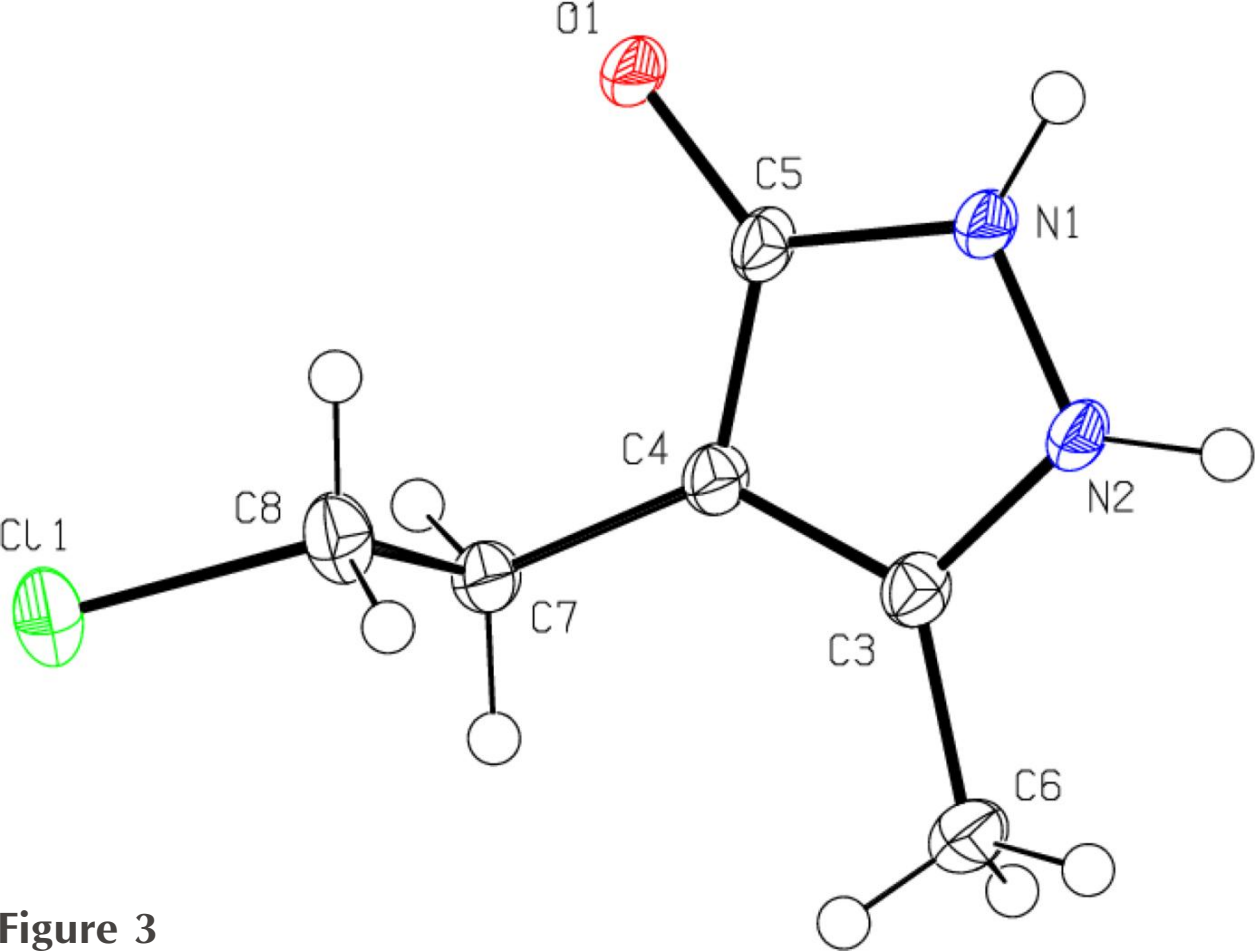
The mol­ecular structure of the title compound, showing the atom labelling and displacement ellipsoids drawn at the 50% probability level.

In the title compound (Fig. 3 ▸), the pyrazoline ring (N1/N2/C3–C5) has an essentially planar conformation [maximum deviation = 0.006 (1) Å for N1]. The C3—C4—C7—C8 and C4—C7—C8—Cl1 torsion angles are 105.67 (19) and 172.38 (11)°, respectively. The geometric parameters of the title compound are normal and comparable to those of related compounds given in the *Database survey* section.

## 3. Supra­molecular features and Hirshfeld surface analysis

**Figure 4:**
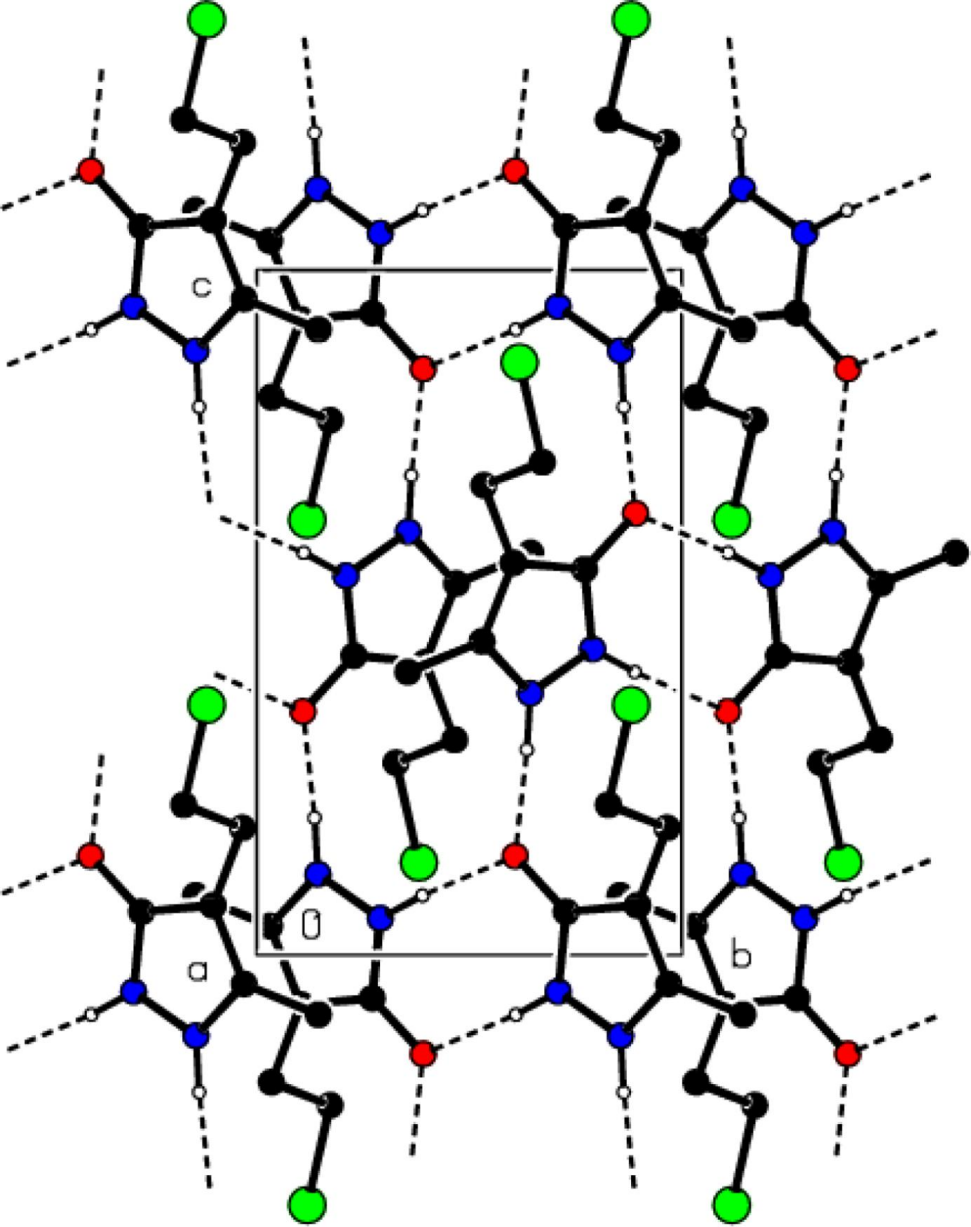
View of the N—H⋯O hydrogen bonds of the title compound down the *a*-axis.

**Figure 5:**
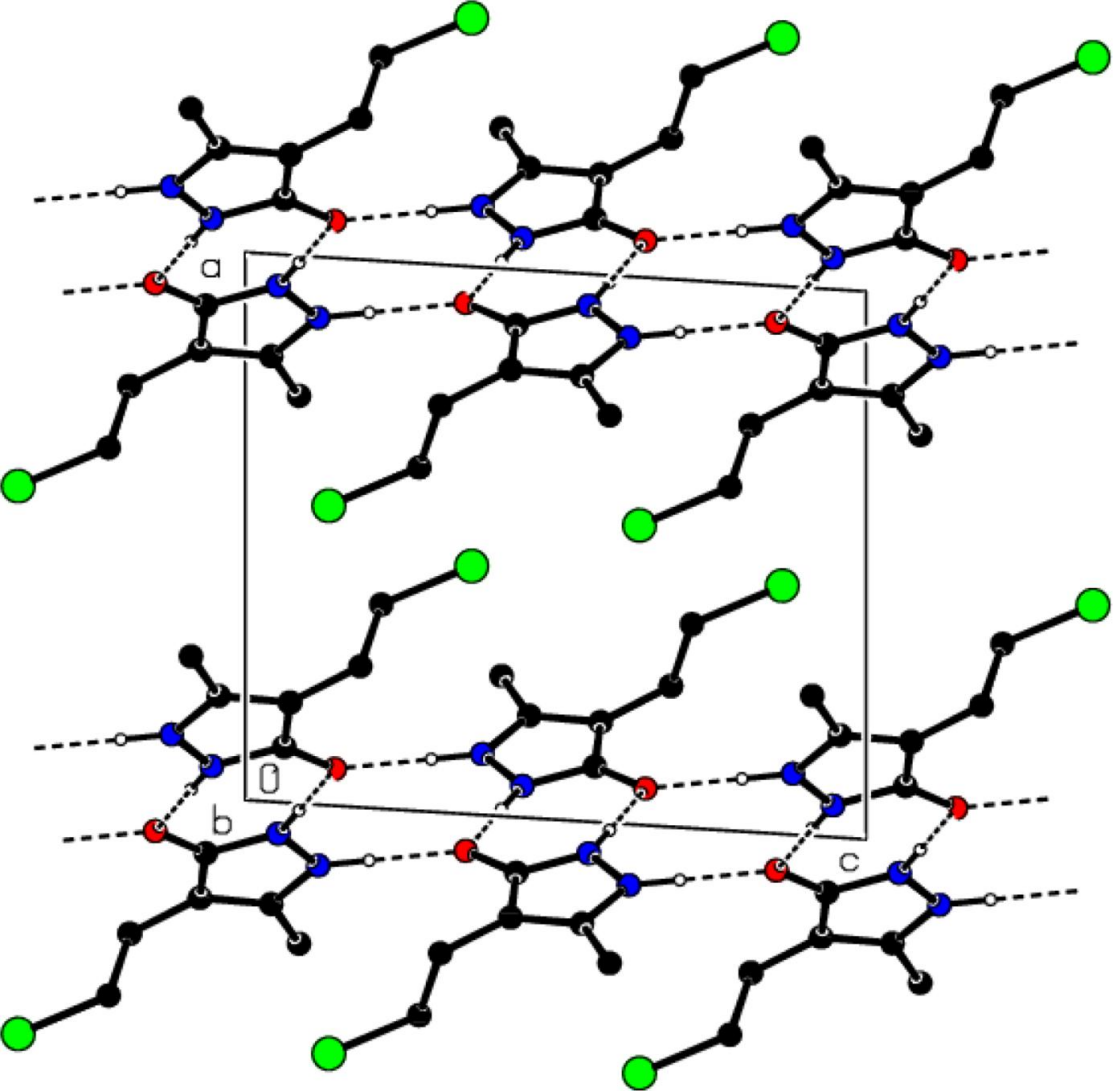
View of the N—H⋯O hydrogen bonds of the title compound down the *b*-axis.

**Figure 7:**
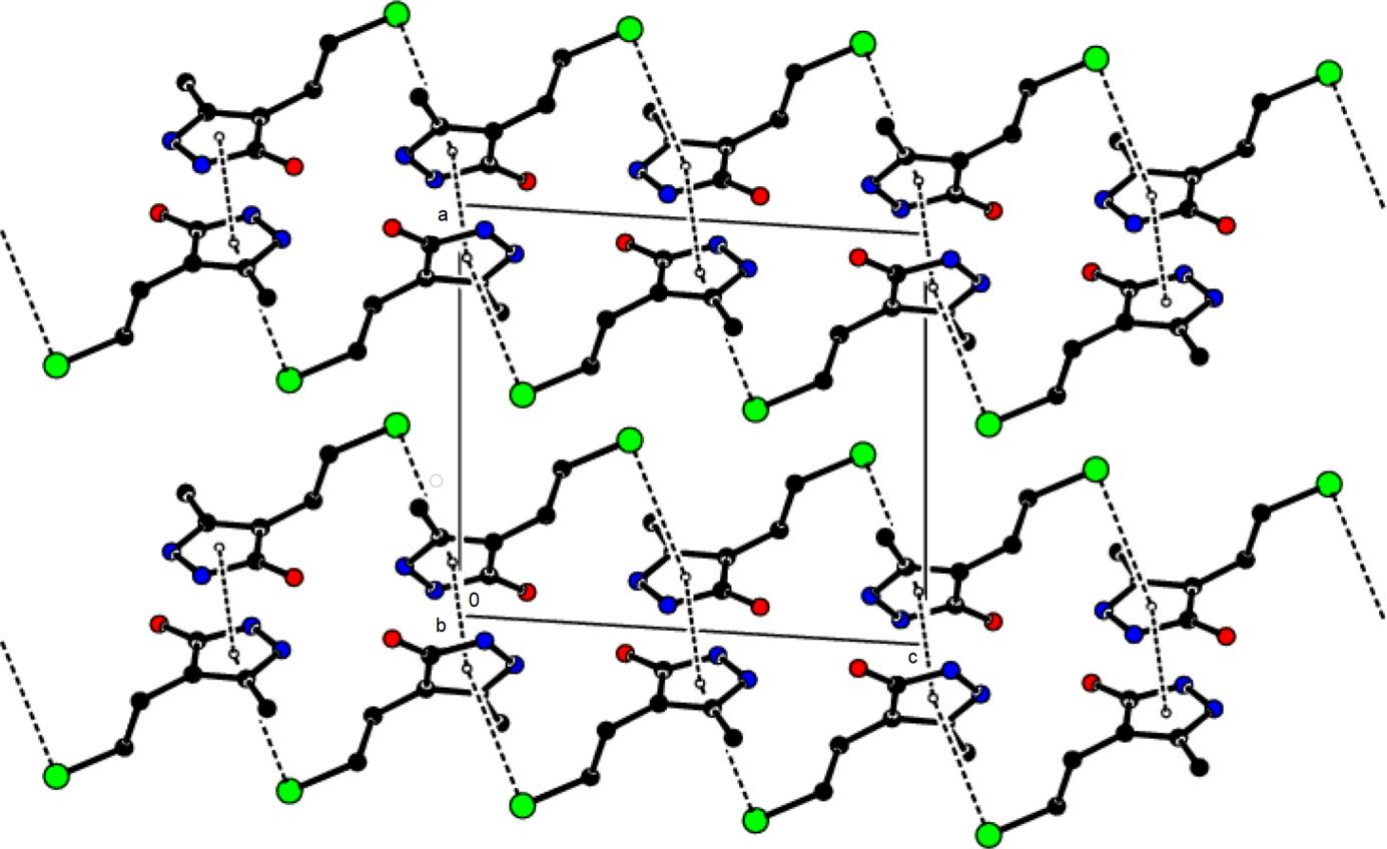
View of the π-π- and C—Cl⋯π inter­actions of the title compound down the *b*-axis.

In the crystal, mol­ecular pairs form dimers with an 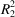 (8) motif (Bernstein *et al.*, 1995 ▸) through N—H⋯O hydrogen bonds (Table 1 ▸ and Fig. 4 ▸). These dimers are also connected into ribbons parallel to the (100) plane by forming N—H⋯O hydrogen bonds with 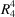 (10) motifs along the *c*-axis direction (Figs. 5 ▸ and 6 ▸). In addition, π–π [*Cg*1⋯*Cg*1^i^ = 3.4635 (9) Å, slippage = 0.511 Å; symmetry code: (i) − *x*, 1 − *y*, 1 − *z*; *Cg*1 is a centroid of the pyrazole ring (N1/N2/C3–C5)] and C—Cl⋯π [C8—Cl1⋯*Cg*1^ii^: C8—Cl1 = 1.8040 (18) Å, Cl1⋯*Cg*1^ii^ = 3.8386 (8) Å, C8—Cl1⋯*Cg*1^ii^ = 84.57 (6)°; symmetry code: (ii) *x*, 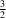 − *y*, 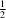 + *z*] inter­actions between the ribbons form layers parallel to the (100) plane. The three-dimensional consolidation of the crystal structure is also ensured by the Cl⋯H and Cl⋯Cl inter­actions [(C8)Cl1⋯H6*B* ^iii^ = 3.12 (3) Å, C8—Cl1⋯H6*B* ^iii^ = 135.3 (6)° and (C8)Cl1⋯Cl1^iv^ = 3.5071 (7) Å, C8—Cl1⋯Cl1^iv^ = 161.79 (7)°; symmetry codes: (iii) 1 − *x*, 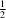 + *y*, 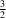 − *z*; (iv) 1 − *x*, 1 − *y*, 2 − *z*] between these layers (Table 2 ▸; Fig. 7 ▸).

**Figure 8:**
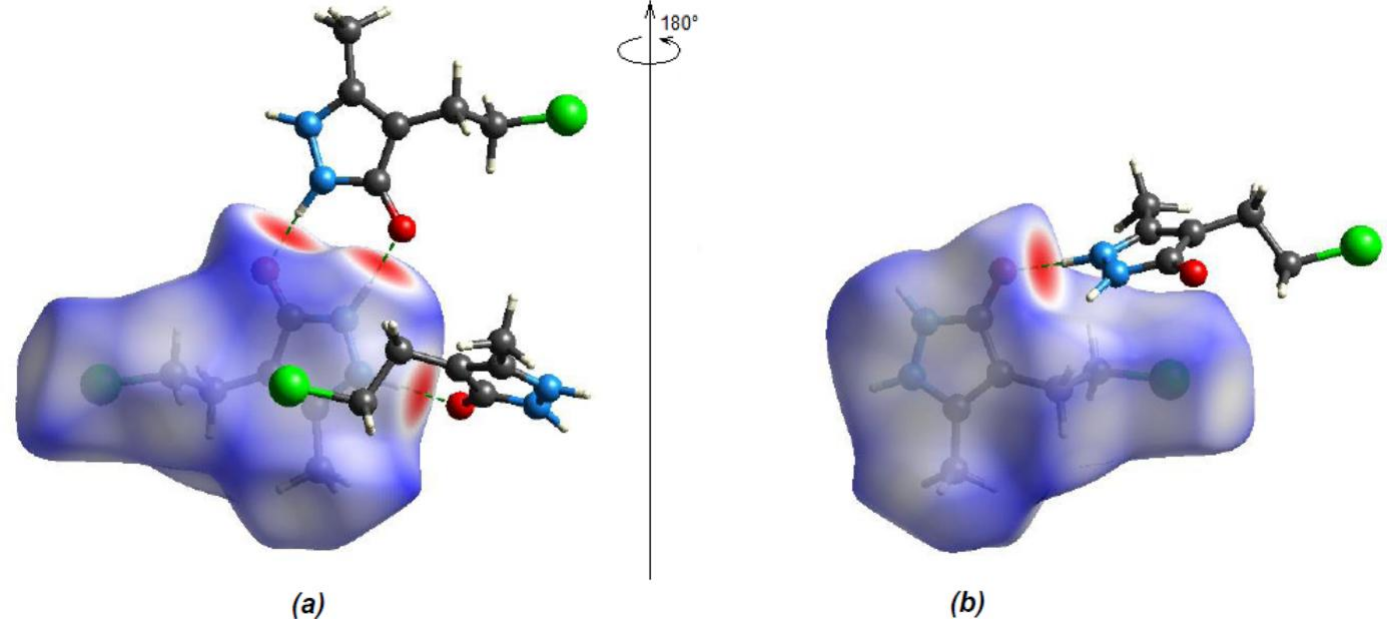
(*a*) Front and (*b*) back sides of the three-dimensional Hirshfeld surface of the title compound mapped over *d*_norm_, with a fixed colour scale of −0.7296 to +1.3271 a.u.

**Figure 9:**
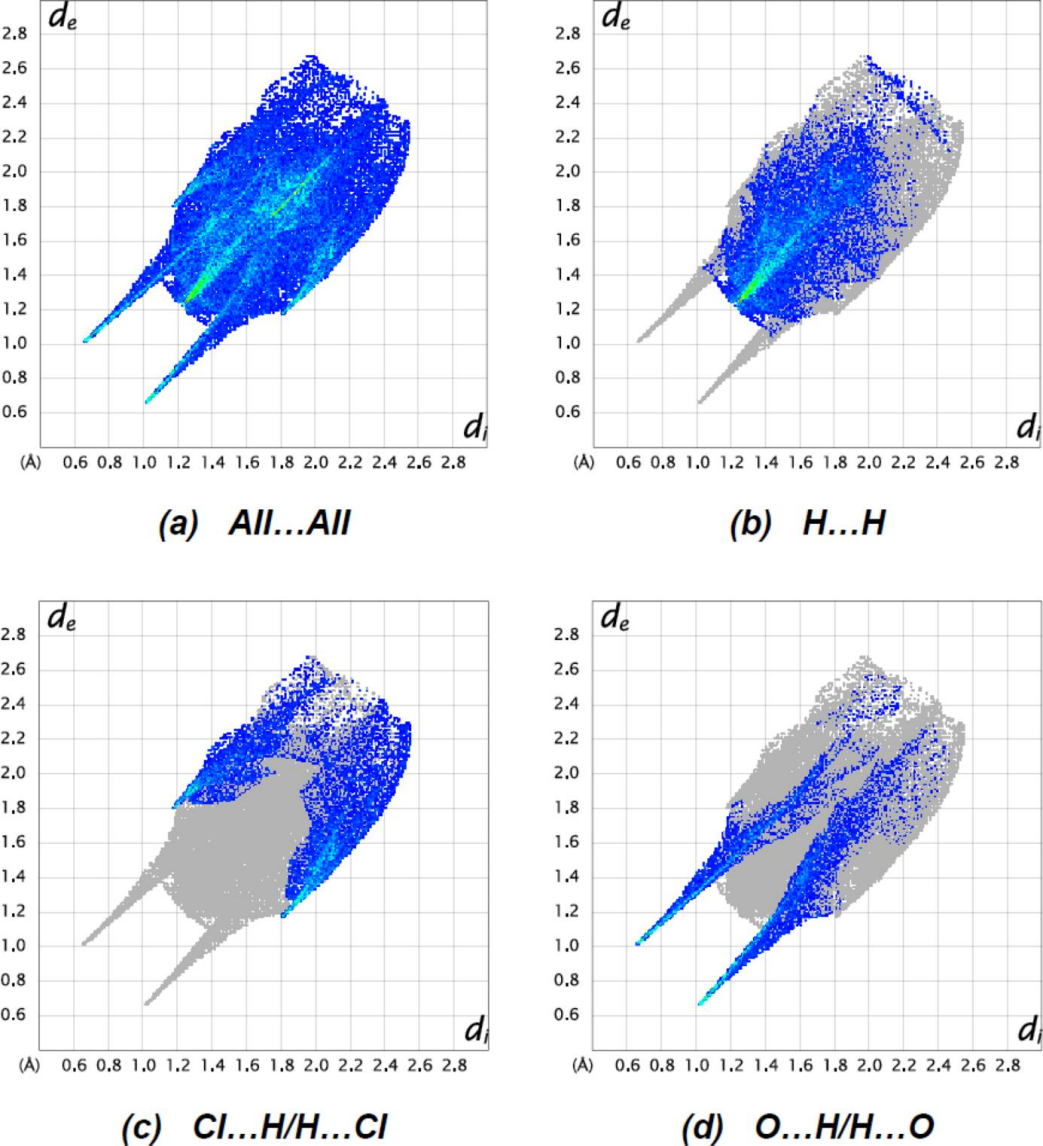
The two-dimensional fingerprint plots of the title compound, showing (*a*) all inter­actions, and delineated into (*b*) H⋯H, (*c*) Cl⋯H/H⋯Cl and (*d*) O⋯H/H⋯O inter­actions. [*d*_e_ and *d*_i_ represent the distances from a point on the Hirshfeld surface to the nearest atoms outside (external) and inside (inter­nal) the surface, respectively].

To qu­antify the inter­molecular inter­actions in the crystal, two-dimensional fingerprint plots and Hirshfeld surfaces were produced using *Crystal Explorer 17.5* (Spackman *et al.*, 2021 ▸). Fig. 8 ▸ shows the mapping of the Hirshfeld surfaces over *d*_norm_ in the range −0.7296 (red) to +1.3271 (blue) a.u. The inter­actions given in Tables 1 ▸ and 2 ▸ play a key role in the mol­ecular packing of the title compound. H⋯H is the most significant inter­atomic contact because it contributes the most to the crystal packing (43.3%, Fig. 9 ▸ *b*). Other significant contributions are made by Cl⋯H/H⋯Cl (22.1%, Fig. 9 ▸ *c*) and O⋯H/H⋯O (18.7%, Fig. 9 ▸ *d*) inter­actions. The following inter­actions make minor contributions: Cl⋯C/C⋯Cl (2.4%), C⋯H/H⋯C (2.6%), N⋯H/H⋯N (4.3%), N⋯C/C⋯N (3.4%), Cl⋯N/N⋯Cl (0.7%), and C⋯C (0.7%).

## 4. Database survey

A search of the Cambridge Structural Database (CSD, Version 5.43, last update November 2022; Groom *et al.*, 2016 ▸) for the central five-membered ring *2,3-di­hydro-1H-pyrazole* yielded six compounds related to the title compound, *viz*. 3-methyl-5-(3-methyl­phen­oxy)-1-phenyl-1*H*-pyrazole-4-carb­aldehyde (CSD refcode TERZAV; Archana, *et al.*, 2022 ▸), *N*-{3-cyano-1-[2,6-di­chloro-4-(tri­fluoro­meth­yl)phen­yl]-4-(ethyl­sulf­an­yl)-1*H*-pyrazol-5-yl}-2,2,2-tri­fluoro­acetamide (FERPOL; Priyanka *et al.*, 2022 ▸), 4-[3-(4-hy­droxy­phen­yl)-4,5-di­hydro-1*H*-pyrazol-5-yl]-2-meth­oxy­phenol monohydrate (KOXGAI; Duong Khanh *et al.*, 2019 ▸), 5-chloro-*N* ^1^-(5-phenyl-1*H*-pyrazol-3-yl)benzene-1,2-di­amine (CAXZUZ; Yartsev *et al.*, 2017 ▸), 5-(butyl­amino)-3-methyl-1-(pyridin-2-yl)-1*H*-pyrazole-4-carbaldehyde (EYEHEX; Macías *et al.*, 2016 ▸) and 5-amino-1-(2-chloro­phen­yl)-1*H*-pyrazole-4-carbo­nitrile (AFIJOP; Lin *et al.*, 2007 ▸).

The mol­ecular packing of TERZAV features aromatic π–π stacking and weak C—H⋯π inter­actions. In the crystal of FERPOL, strong N—H⋯O hydrogen bonds link the mol­ecules into chains that extend parallel to the *a*-axis. In the crystal of KOXGAI, the mol­ecules are connected into chains running in the *b*-axis direction by O—H⋯N hydrogen bonding. Parallel chains inter­act through N—H⋯O hydrogen bonds and π–π stacking of the tris­ubstituted phenyl rings. In the crystal of CAXZUZ, the *A* and *B* mol­ecules are linked by two pairs of N—H⋯N hydrogen bonds, forming *A*–*B* dimers. These are further linked by a fifth N—H⋯N hydrogen bond, forming tetra­mer-like units that stack along the *a*-axis direction, forming columns, which are in turn linked by C—H⋯π inter­actions, forming layers parallel to the *ac* plane. The supra­molecular structure of EYEHEX assembly has a three-dimensional arrangement controlled mainly by weak C—H⋯O and C—H⋯π inter­actions. The crystal structure of AFIJOP is consolidated by two N—H⋯N hydrogen bonds.

## 5. Synthesis and crystallization

Aceto­acetic ether (7.7 mmol), di­chloro­ethane (7.7 mmol) and hydrazine hydrate (15.4 mmol) were dissolved in 40 ml of ethanol and the reaction mixture was refluxed for 4 h. Then the reaction mixture was cooled to room temperature with the formation of white crystals. The crystals were separated by filtration and recrystallized from an ethanol–water mixture (m.p. 499–500 K, yield 78%).

^1^H NMR (300 MHz, DMSO-*d*_6_, ppm.): 2.06 (*s*, 3H, CH_3_); 2.64 (*t*, 2H, CH_2_, ^H-H^ *J*_2_ = 7.2); 3.49 (*s*, 2H, 2NH); 3.58 (*t*, 2H, ClCH_2_, ^H-H^ *J*_2_ = 7.2). ^13^C NMR (75 MHz, DMSO-*d*_6_, ppm.): 10.28 (CH_3_), 26.02 (CH_2_), 44.91 (CH_2_Cl), 97.63 (C_*tert.*_=), 160.12 (HN—C_*tert.*_=), 162.34 (N—C=O).

## 6. Refinement

Crystal data, data collection and structure refinement details are summarized in Table 3 ▸. The C-bound H atoms were placed in calculated positions (0.95–0.99 Å) and refined as riding with *U*_iso_(H) = 1.2 or 1.5*U*_eq_(C). The N-bound H atoms were located in a difference map and freely refined.

## Supplementary Material

Crystal structure: contains datablock(s) I. DOI: 10.1107/S2056989024000835/nx2004sup1.cif

Structure factors: contains datablock(s) I. DOI: 10.1107/S2056989024000835/nx2004Isup2.hkl

Supporting information file. DOI: 10.1107/S2056989024000835/nx2004Isup3.cml

CCDC reference: 2327646

Additional supporting information:  crystallographic information; 3D view; checkCIF report

## Figures and Tables

**Figure 6:**
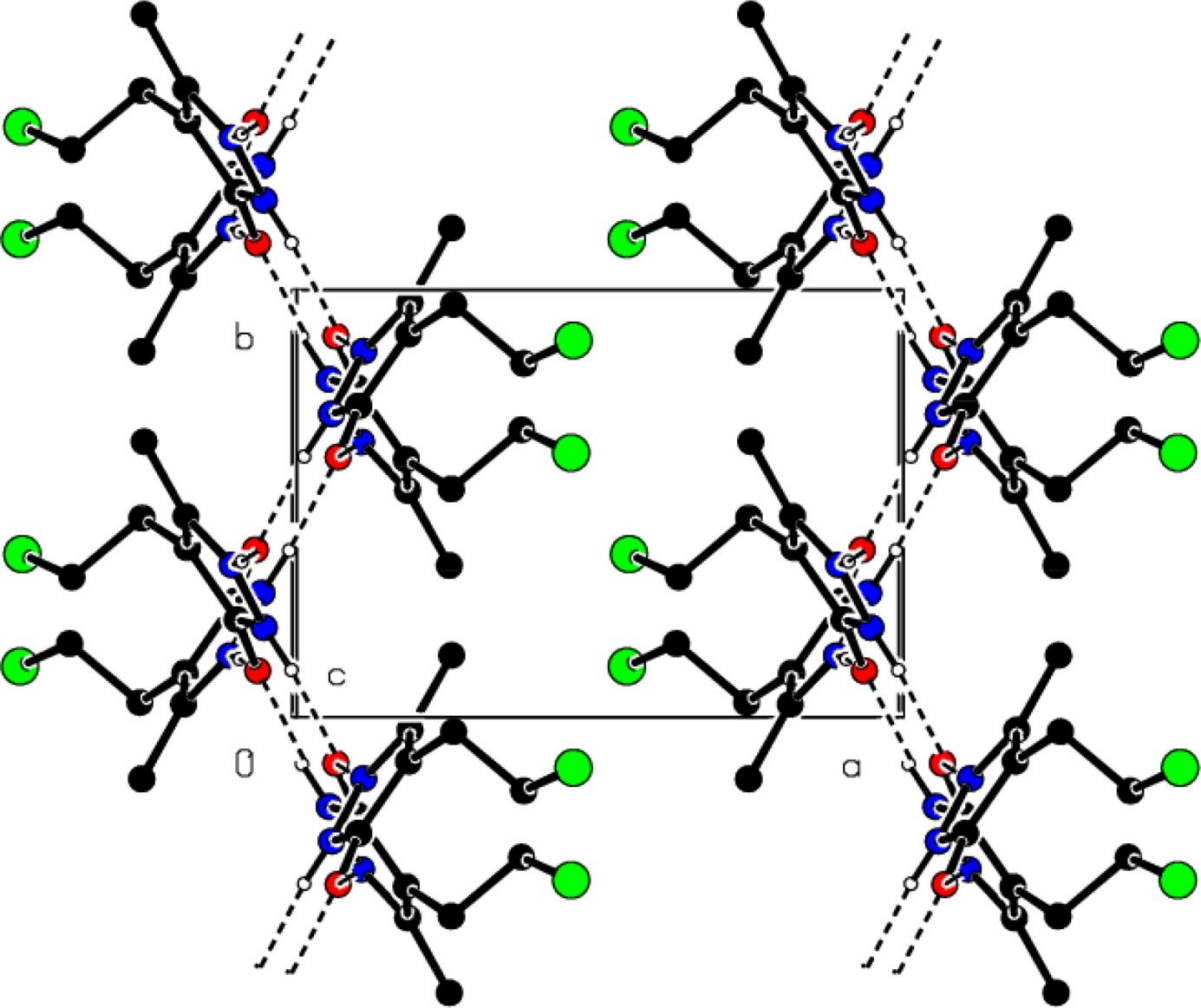
View of the N—H⋯O hydrogen bonds of the title compound down the *c*-axis.

**Table 1:** Hydrogen-bond geometry (Å, °)

*D*—H⋯*A*	*D*—H	H⋯*A*	*D*⋯*A*	*D*—H⋯*A*
N1—H1⋯O1^i^	0.88 (3)	1.81 (3)	2.6861 (18)	174 (2)
N2—H2⋯O1^ii^	0.92 (3)	1.75 (3)	2.6772 (17)	177 (2)

Symmetry codes: (i) 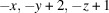 ; (ii) 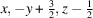 .

**Table 2:** Summary of short inter­atomic contacts (Å) in the title compound

Cl1⋯H6*B*	3.12	1 − *x*, 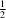 + *y*, 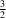 − *z*
Cl1⋯Cl1	3.51	1 − *x*, 1 − *y*, 2 − *z*
H1⋯O1	1.80	−*x*, 2 − *y*, 1 − *z*
H6*C*⋯O1	2.89	−*x*, 1 − *y*, 1 − *z*
O1⋯H2	1.76	*x*, 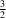 − *y*, 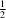 + *z*
H6*A*⋯H7*B*	2.60	*x*, 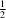 − *y*, − 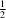 + *z*

**Table 3:** Experimental details

Crystal data	
Chemical formula	C_6_H_9_ClN_2_O
*M*_r_	160.60
Crystal system, space group	Monoclinic, *P*2_1_/*c*
Temperature (K)	100
*a*, *b*, *c* (Å)	9.8420 (2), 6.9145 (2), 11.1807 (2)
β (°)	93.618 (2)
*V* (Å^3^)	759.36 (3)
*Z*	4
Radiation type	Cu *K*α
μ (mm^−1^)	3.92
Crystal size (mm)	0.20 × 0.12 × 0.06

Data collection	
Diffractometer	XtaLAB Synergy, Dualflex, HyPix
Absorption correction	Multi-scan (*CrysAlis PRO*; Rigaku OD, 2022 ▸)
*T*_min_, *T*_max_	0.513, 0.750
No. of measured, independent and observed [*I* > 2σ(*I*)] reflections	6642, 1532, 1467
*R*_int_	0.027
(sin θ/λ)_max_ (Å^−1^)	0.633

Refinement	
*R*[*F* ^2^ > 2σ(*F* ^2^)], *wR*(*F* ^2^), *S*	0.036, 0.097, 1.05
No. of reflections	1532
No. of parameters	127
H-atom treatment	All H-atom parameters refined
Δρ_max_, Δρ_min_ (e Å^−3^)	0.28, −0.41

Computer programs: *CrysAlis PRO* (Rigaku OD, 2022 ▸), *SHELXT* (Sheldrick, 2015*a* ▸), *SHELXL* (Sheldrick, 2015*b* ▸), *ORTEP-3 for Windows* (Farrugia, 2012 ▸) and *PLATON* (Spek, 2020 ▸).

## supplementary crystallographic information

### Crystal data

**Table:**

C_6_H_9_ClN_2_O	*F*(000) = 336
*M**_r_* = 160.60	*D*_x_ = 1.405 Mg m^−^^3^
Monoclinic, *P*2_1_/*c*	Cu *K*α radiation, λ = 1.54184 Å
*a* = 9.8420 (2) Å	Cell parameters from 4592 reflections
*b* = 6.9145 (2) Å	θ = 4.5–77.6°
*c* = 11.1807 (2) Å	µ = 3.92 mm^−^^1^
β = 93.618 (2)°	*T* = 100 K
*V* = 759.36 (3) Å^3^	Prism, colourless
*Z* = 4	0.20 × 0.12 × 0.06 mm

### Data collection

**Table:**

XtaLAB Synergy, Dualflex, HyPix diffractometer	1467 reflections with *I* > 2σ(*I*)
Radiation source: micro-focus sealed X-ray tube	*R*_int_ = 0.027
ω scans	θ_max_ = 77.5°, θ_min_ = 4.5°
Absorption correction: multi-scan (CrysAlisPro; Rigaku OD, 2022)	*h* = −12→12
*T*_min_ = 0.513, *T*_max_ = 0.750	*k* = −8→7
6642 measured reflections	*l* = −14→8
1532 independent reflections	

### Refinement

**Table:**

Refinement on *F*^2^	Primary atom site location: difference Fourier map
Least-squares matrix: full	Secondary atom site location: difference Fourier map
*R*[*F*^2^ > 2σ(*F*^2^)] = 0.036	Hydrogen site location: difference Fourier map
*wR*(*F*^2^) = 0.097	All H-atom parameters refined
*S* = 1.05	*w* = 1/[σ^2^(*F*_o_^2^) + (0.0501*P*)^2^ + 0.606*P*] where *P* = (*F*_o_^2^ + 2*F*_c_^2^)/3
1532 reflections	(Δ/σ)_max_ = 0.001
127 parameters	Δρ_max_ = 0.28 e Å^−^^3^
0 restraints	Δρ_min_ = −0.41 e Å^−^^3^

### Special details

**Table:**

Geometry. All esds (except the esd in the dihedral angle between two l.s. planes) are estimated using the full covariance matrix. The cell esds are taken into account individually in the estimation of esds in distances, angles and torsion angles; correlations between esds in cell parameters are only used when they are defined by crystal symmetry. An approximate (isotropic) treatment of cell esds is used for estimating esds involving l.s. planes.

### Fractional atomic coordinates and isotropic or equivalent isotropic displacement parameters (Å^2^)

**Table:**

	*x*	*y*	*z*	*U*_iso_*/*U*_eq_	
Cl1	0.45324 (5)	0.61848 (7)	0.86496 (4)	0.03447 (18)	
O1	0.06705 (13)	0.89161 (17)	0.64445 (10)	0.0231 (3)	
N1	0.05777 (14)	0.7905 (2)	0.44644 (12)	0.0197 (3)	
H1	0.013 (3)	0.890 (4)	0.413 (2)	0.040 (7)*	
N2	0.11129 (14)	0.6437 (2)	0.38217 (12)	0.0195 (3)	
H2	0.096 (2)	0.636 (3)	0.300 (2)	0.038 (6)*	
C3	0.18687 (16)	0.5302 (2)	0.45765 (14)	0.0189 (3)	
C4	0.18362 (16)	0.6035 (2)	0.57245 (14)	0.0181 (3)	
C5	0.10154 (16)	0.7717 (2)	0.56311 (13)	0.0188 (3)	
C6	0.2560 (2)	0.3553 (3)	0.41307 (16)	0.0254 (4)	
H6A	0.282 (3)	0.372 (4)	0.331 (3)	0.058 (8)*	
H6B	0.338 (3)	0.328 (5)	0.458 (3)	0.065 (9)*	
H6C	0.205 (3)	0.250 (5)	0.414 (3)	0.066 (9)*	
C7	0.25721 (17)	0.5342 (2)	0.68559 (14)	0.0205 (3)	
H7A	0.194 (2)	0.527 (3)	0.7508 (18)	0.021 (5)*	
H7B	0.295 (2)	0.406 (3)	0.676 (2)	0.029 (5)*	
C8	0.37260 (18)	0.6724 (3)	0.71941 (16)	0.0254 (4)	
H8A	0.340 (2)	0.807 (4)	0.724 (2)	0.031 (6)*	
H8B	0.443 (2)	0.665 (4)	0.664 (2)	0.037 (6)*	

### Atomic displacement parameters (Å^2^)

**Table:**

	*U*^11^	*U*^22^	*U*^33^	*U*^12^	*U*^13^	*U*^23^
Cl1	0.0401 (3)	0.0366 (3)	0.0249 (3)	0.00431 (18)	−0.01242 (19)	−0.00157 (17)
O1	0.0374 (7)	0.0209 (6)	0.0110 (5)	0.0082 (5)	0.0007 (5)	−0.0010 (4)
N1	0.0297 (7)	0.0180 (7)	0.0114 (6)	0.0045 (5)	0.0008 (5)	−0.0006 (5)
N2	0.0278 (7)	0.0194 (7)	0.0115 (7)	0.0026 (5)	0.0013 (5)	−0.0028 (5)
C3	0.0237 (7)	0.0179 (7)	0.0153 (7)	−0.0011 (6)	0.0027 (6)	0.0008 (6)
C4	0.0238 (7)	0.0177 (7)	0.0128 (7)	0.0008 (6)	0.0024 (6)	0.0017 (6)
C5	0.0273 (8)	0.0183 (7)	0.0110 (7)	−0.0002 (6)	0.0023 (6)	0.0003 (6)
C6	0.0333 (9)	0.0233 (8)	0.0198 (9)	0.0057 (7)	0.0033 (7)	−0.0036 (7)
C7	0.0280 (8)	0.0192 (8)	0.0144 (8)	0.0036 (6)	0.0015 (6)	0.0016 (6)
C8	0.0264 (8)	0.0315 (9)	0.0180 (8)	0.0006 (7)	−0.0019 (6)	0.0029 (7)

### Geometric parameters (Å, º)

**Table:**

Cl1—C8	1.8040 (18)	C4—C7	1.496 (2)
O1—C5	1.2920 (19)	C6—H6A	0.97 (3)
N1—C5	1.354 (2)	C6—H6B	0.95 (3)
N1—N2	1.3685 (19)	C6—H6C	0.88 (3)
N1—H1	0.88 (3)	C7—C8	1.514 (2)
N2—C3	1.342 (2)	C7—H7A	0.99 (2)
N2—H2	0.92 (3)	C7—H7B	0.97 (2)
C3—C4	1.382 (2)	C8—H8A	0.99 (2)
C3—C6	1.489 (2)	C8—H8B	0.95 (2)
C4—C5	1.416 (2)		
			
C5—N1—N2	108.95 (13)	H6A—C6—H6B	105 (2)
C5—N1—H1	126.8 (17)	C3—C6—H6C	113 (2)
N2—N1—H1	123.6 (17)	H6A—C6—H6C	107 (3)
C3—N2—N1	108.66 (13)	H6B—C6—H6C	107 (3)
C3—N2—H2	129.9 (15)	C4—C7—C8	108.91 (14)
N1—N2—H2	121.4 (15)	C4—C7—H7A	110.2 (12)
N2—C3—C4	108.96 (14)	C8—C7—H7A	110.3 (12)
N2—C3—C6	120.74 (15)	C4—C7—H7B	111.5 (13)
C4—C3—C6	130.29 (15)	C8—C7—H7B	108.6 (13)
C3—C4—C5	106.22 (14)	H7A—C7—H7B	107.3 (18)
C3—C4—C7	128.97 (15)	C7—C8—Cl1	112.04 (12)
C5—C4—C7	124.69 (14)	C7—C8—H8A	111.5 (13)
O1—C5—N1	122.31 (15)	Cl1—C8—H8A	105.7 (13)
O1—C5—C4	130.49 (14)	C7—C8—H8B	111.5 (15)
N1—C5—C4	107.19 (13)	Cl1—C8—H8B	106.1 (15)
C3—C6—H6A	111.6 (17)	H8A—C8—H8B	109.7 (19)
C3—C6—H6B	111.9 (19)		
			
C5—N1—N2—C3	0.98 (18)	N2—N1—C5—C4	−1.24 (18)
N1—N2—C3—C4	−0.30 (18)	C3—C4—C5—O1	179.99 (17)
N1—N2—C3—C6	178.79 (15)	C7—C4—C5—O1	−3.7 (3)
N2—C3—C4—C5	−0.45 (18)	C3—C4—C5—N1	1.04 (18)
C6—C3—C4—C5	−179.43 (17)	C7—C4—C5—N1	177.36 (15)
N2—C3—C4—C7	−176.56 (16)	C3—C4—C7—C8	105.67 (19)
C6—C3—C4—C7	4.5 (3)	C5—C4—C7—C8	−69.8 (2)
N2—N1—C5—O1	179.70 (15)	C4—C7—C8—Cl1	172.38 (11)

### Hydrogen-bond geometry (Å, º)

**Table:**

*D*—H···*A*	*D*—H	H···*A*	*D*···*A*	*D*—H···*A*
N1—H1···O1^i^	0.88 (3)	1.81 (3)	2.6861 (18)	174 (2)
N2—H2···O1^ii^	0.92 (3)	1.75 (3)	2.6772 (17)	177 (2)

Symmetry codes: (i) −*x*, −*y*+2, −*z*+1; (ii) *x*, −*y*+3/2, *z*−1/2.
